# Effects of Glossopharyngeal Insufflation in Ankylosing Spondylitis: A Pilot Study

**DOI:** 10.1155/2014/594708

**Published:** 2014-11-23

**Authors:** Nina Brodin, Peter Lindholm, Claudia Lennartsson, Malin Nygren-Bonnier

**Affiliations:** ^1^Department of Neurobiology, Care Sciences and Society, Division of Physiotherapy, Karolinska Institutet, B3, Huddinge, 14183 Stockholm, Sweden; ^2^Department of Orthopaedics, Division of Physiotherapy, Danderyd Hospital, 182 88 Stockholm, Sweden; ^3^Department of Radiology, Karolinska University Hospital, 171 76 Stockholm, Sweden; ^4^Department of Physiology and Pharmacology, Karolinska Institutet, 171 77 Stockholm, Sweden; ^5^Department of Physical Therapy, Karolinska University Hospital, 141 86 Stockholm, Sweden

## Abstract

In Ankylosing Spondylitis (AS), thoracic range of motion is often greatly limited. The objective of the study was to describe the effects of 12 weeks of Glossopharyngeal Insufflation (GI) training in patients with AS. Dynamic spirometry included vital capacity, forced expiratory volume, and peak expiratory flow. Thoracic and lumbar range of motion was assessed by tragus-to-wall distance, modified Schober test, and tape measure. Disease activity, activity limitation, and health perception were assessed using the BAS-Indices, and tension in the thoracic region during GI was assessed using the Borg CR-10 scale. Adherence to training was recorded in an activity log, along with any remarks on the training. Ten patients were recruited and six male patients fulfilled the study protocol. Three patients were able to learn GI by exceeding their maximal vital capacity with 5% using GI. A significant increase in thoracic range of motion both on costae IV (*P* = 0.04) and at the level of the xiphoid process (*P* = 0.04) was seen. Thus, patients with AS can practice GI, it is safe if maximal exertion is avoided, and patients with some mobility in the chest can increase their lung function substantially by performing GI during 12 weeks.

## 1. Introduction

Ankylosing Spondylitis (AS) is a chronic inflammatory disease of multifactorial impact to the patient. Pain, stiffness, and fatigue are common symptoms affecting the patient's function, activity, and participation in society [1, 2]. Inflammation mainly affects the spinal joints, which may lead to imbalance in the costotransverse joints with pain and greatly limited thoracic mobility as a result. Due to this, respiratory function can be affected, most often described as restrictive ventilatory impairment [3], indicating that the lung tissue is unaffected, and instead the thorax has reduced ability to expand due to weak respiratory muscles, inflammatory pain, or reduced mobility [4, 5]. This can lead to reduced ventilation, impaired coughing function, secretion stagnation, and even more limited thoracic range of motion, which in turn can lead to serious respiratory complications.

Glossopharyngeal Insufflation (GI) training has been used since the 1950s by patients with reduced lung volume [6]. It is an alternative technique of breathing which maintains adequate ventilation and improves cough function when respiratory muscles are weak [7]. It is also used by breath-hold divers to help them increase lung volume above their normal total lung capacity (TLC) and thereby increase diving performance [8]. This breathing technique is performed by using the glossopharyngeal muscles to insufflate boluses of air into the lungs [9].

The mechanics of GI are described in previous studies [9, 10]. A study by Collier et al. [10] showed that the resistive work during GI is rather small. More effort is required to expand the thorax than the lungs. Previous studies in healthy persons and persons with cervical spinal cord injury have shown that lung function and thoracic range of motion can improve with GI [11–13]. This improvement was suggested as most likely an effect of increased range of motion in the musculoskeletal system and not primarily caused by an increase in pulmonary tissue or increased pulmonary compliance. Even though the individual contributions of these factors have not been evaluated, the indication that the effect is caused by increases in flexibility suggests that patients with decreased flexibility could benefit from GI training. Persons diagnosed with AS fit this description, and no study has evaluated the effect of GI in patients with AS.

Therefore, the primary aim of this pilot study was to describe the effects of GI on thoracic range of motion and secondarily on lung function, lumbar and cervical range of motion, disease activity, activity limitations, and health perception in patients with AS.

## 2. Patients and Methods

### 2.1. Patients

Outpatients at the Physiotherapy Department at the Orthopaedic Clinic, Danderyd Hospital, and at the Physiotherapy Department at the Karolinska University Hospital in Stockholm were eligible for inclusion in the study if age >18 years and diagnosed with AS according to the modified New York criteria [14]. Exclusion criteria were injuries or diseases that could possibly affect the execution or possible effects of GI such as infections with fever or cognitive impairments affecting the ability to understand and learn the technique. According to a power calculation based on change of the primary outcome variable (thoracic range of motion) *P* level of 0.05 and power of 80%, 8 patients would be sufficient. Eleven patients were invited to participate and 10 accepted, signed informed consent, and were included in the study. Ethical approval for the study was given by the regional ethics committee at Karolinska Institutet (Dnr 2009/1195-31/1).

### 2.2. Procedure

Patients were instructed on how to perform the GI technique in practice, using written material and by watching an instruction film. Patients met and practiced GI with one of the two researchers 2-3 times weekly during 2-3 weeks, until they were able to perform the GI technique correctly. By measuring vital capacity (VC) during the GI training (VCGI) it was determined if the patient had learned the technique. This was accomplished when the patient exceeded their baseline VC during GI with any volume. Before the intervention period started, all patients were assessed by one of two researchers and after this, each patient performed GI during 12 weeks, at least 4 times/week and approximately 15 minutes each time. The rationale for this was based upon previous studies indicating that this amount of exercise is sufficient to reach effects in both range of motion and lung function GI [11, 13]. After 12 weeks of GI training, all patients were assessed again by one of the two researchers.

### 2.3. Intervention

In glossopharyngeal breathing, the patients first carry out a maximal inhalation; the tongue is then pushed upwards and backwards forcing the air into the pharynx. The larynx opens, and the air passes into the trachea where it is trapped by closure of the larynx [9], followed by the relaxing of the larynx, when the air is passively expelled [15]. This action is the mechanism of each gulp. A gulp is defined as the boluses of air projected into the trachea by the pistoning action of the tongue [7, 10, 16]. This cycle is repeated, using as many gulps of air as possible without discomfort. All patients carried out a short warm-up with stretching exercises for the chest before starting their training session. After this, they performed 10 repetitions of GI in a sitting or supine position. Every 3-4 week, a physiotherapist controlled that the technique was adequately performed by taking measurements of lung function. The patients then performed GI on their own, at home.

### 2.4. Assessments

All patients were instructed to fill in a personal activity log for the 12-week study period. Information on when GI was performed, number of gulps per cycle, number of cycles, perceived tension in the chest during GI using the Borg CR-10 scale [17], and any remarks on the training were recorded.

Lung function test with dynamic spirometry was performed before and after the intervention and included measurements of VC, forced expiratory volume in one second (FEV_1_) and peak expiratory flow (PEF), in accordance with current American Thoracic Society standard [18]. A portable infrared interruption flow sensor (Micro Loop; Cardinal Health, Basingstoke, UK) was used and calibrated before each assessment.

Thoracic range of motion was assessed by calculating the difference in range of motion at the level of the fourth costae and the xiphoid process, respectively, during maximal inhalation and exhalation using a tape measure. The patients were instructed to perform a maximal exhalation (to RV) followed by an inhalation to TLC [19].

Cervical and lumbar range of motion (ROM) was assessed by tragus-to-wall distance (cm) and the modified Schober test using a tape measure following the instructions in the Bath Ankylosing Spondylitis Metrology Index [20]. Further, disease activity was assessed by the Bath Ankylosing Spondylitis Disease Activity Index (BASDAI) ranging from 0 to 10, where 0 reflects no disease activity and 10 maximal disease activity [21].

Activity limitation was assessed by the Bath Ankylosing Spondylitis Functional Index (BASFI) ranging from 0 to 10, where 0 indicates no activity limitations and 10 maximal activity limitation [22].

Health perception of the past week was assessed using the Bath Ankylosing Spondylitis Global Index (BAS-G). The range of the BAS-G is 0–10, with 0 indicating best possible health and 10 indicating worst possible health [23].

### 2.5. Statistics

Due to the low number of observations, nonparametric statistical methods were used. Descriptive statistics are presented as median and range. Wilcoxon matched pairs test were used to analyze effects of GI on primary and secondary outcome measures. The STATISTICA software (version 12.0, Stat Soft Inc., Tulsa, OK, USA) was used.

## 3. Results

Of the ten patients participating in baseline assessment, four patients dropped out during the study period; two dropped out due to increased occupational workload, one moved abroad, and one discontinued participation without giving a reason. Ultimately, six male patients fulfilled the study protocol. For baseline characteristics of the six patients, see Table 1. Three of the six patients (Cases 1, 4, and 6) were able to learn GI as they exceeded their maximal VC with 5% using GI (Table 2). They increased their VC with GI by, 1.57, 0.43, and 1.89 l, respectively.

**Figure 1:**
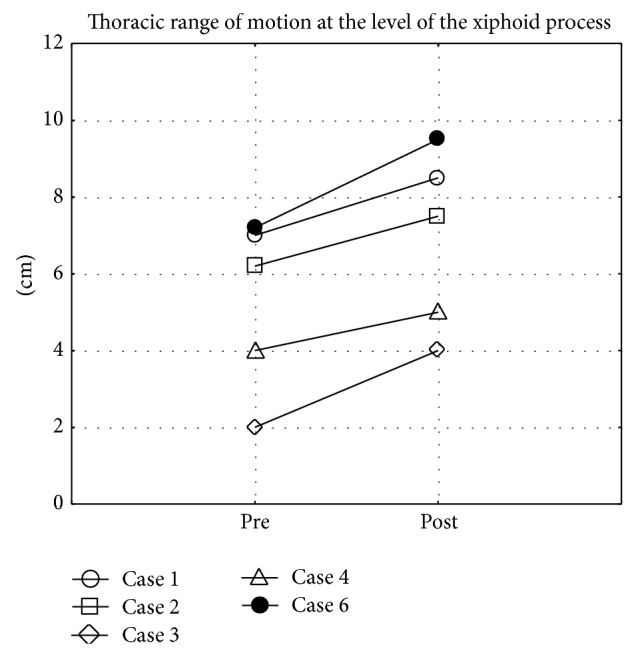
Difference in thoracic range of motion between maximal inhalation and exhalation at the level of the xiphoid process before (pre) and after (post) 12 weeks of Glossopharyngeal Insufflation (missing data on 1 subject).

**Figure 2:**
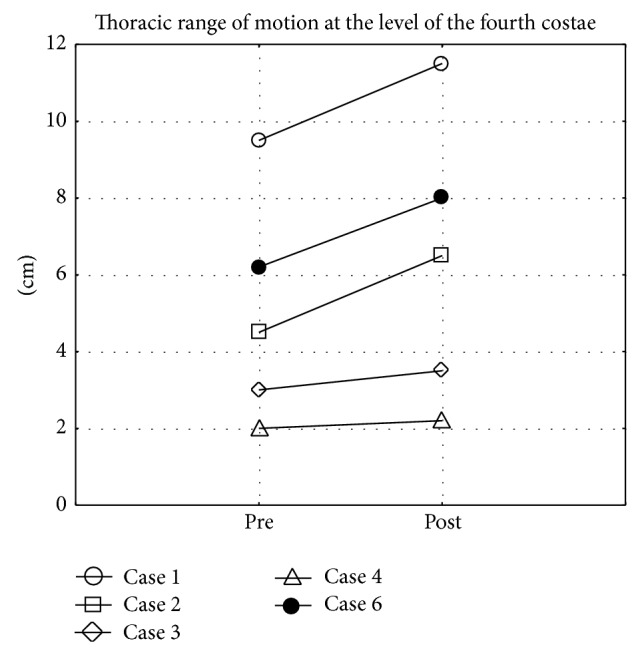
Difference in thoracic range of motion between maximal inhalation and exhalation at the level of the fourth costae before (pre) and after (post) 12 weeks of Glossopharyngeal Insufflation (missing data on 1 subject).

There was a statistically significant increase in thoracic range of motion at the level of the xiphoid process (*P* = 0.04) (Figure 1) and of the fourth costae (*P* = 0.04) (Figure 2) before and after 12 weeks of GI training. There were no significant differences in other range of motion parameters, disease activity, activity limitation, or perceived health. All individual results from baseline and after 12 weeks are presented in Table 2.

Three of the patients occasionally reported temporary adverse symptoms such as tension in the chest and fatigue occurring during or shortly after performing GI, while three reported no adverse symptoms. At assessment postintervention, one patient demonstrated extreme dizziness and local paresthesia which subsided after 8–10 minutes.

## 4. Discussion

The main findings were that thoracic range of motion increased, probably due to stretching induced by GI.

Only three of the six patients learned GI properly by exceeding the maximal VC over 5%. This could be explained by several factors, such as lack of motivation, insufficient instruction, too short a period to learn GI, or physiological factors as an already too stiff chest. Dickinson et al. [24] showed that motivation and proper instruction are important aspects of the ability to learn the GI technique, and possibly the instructors were too inexperienced in judging if the technique was properly applied, thus leading to the intervention period starting too early. The researchers responsible for teaching the participants the GI technique were very experienced within rheumatology, but not within lung medicine, and might thus have had too little experience of the technique to be able to teach it to all participants properly. However, both assessors were educated in GI, assessment of lung function and handling of the Micro Loop prior to study start, in an attempt to minimize such effects. This pilot study also had further limitations. The number of participants was small and after dropouts power was no longer met, leading to a lower possibility of correctly describing the effects of GI in patients with AS. Also, we were not able to recruit any women with AS fulfilling the study protocol, and because of this we cannot say anything about the effects of GI in women. The fact that 40% of the baseline assessed participants did not fulfill the study protocol needs to be commented on. To do the GI exercises for approximately 15 minutes, at a minimum of four times per week for 12 weeks, might seem as an acceptable workload. However, these patients regularly perform aerobic and strengthening exercises and most of them probably also perform daily range of motion exercises, and to perform the GI exercises on top of this might be considered too much for some participants, as discussed earlier.

Two of the cases with VCGI over 1.5 l also had the largest thoracic range of motion at baseline. This might suggest that GI is more useful in patients who still have some mobility in the chest wall and thus might increase their VC by 30% when using GI. All lung function parameters increased noticeably in these two subjects, further consolidating that they had performed the technique properly. Thoracic range of motion increased significantly in all participants after the training period of GI. With GI, a substantial volume of gas is added to the lungs, and the major effect of the added gas is an expansion of the chest. This chest expansion, beyond “normal,” results in a stretching effect of the respiratory system, which has also been shown in previous studies in healthy subjects [11, 12]. Also, the participants who did not learn GI completely seemed to have effects on thoracic range of motion by performing maximal GI maneuvers several times a week. This might be explained as a result of stretching the chest wall and the structures surrounding the rib cage and is also in line with earlier studies examining the effects of breathing exercises as physiotherapeutic treatment on range of motion in AS [25–28]. Range of motion exercise is very common in AS to maintain or improve joint mobility, especially in the thoracic region. However, these exercises are commonly performed by bending or turning the torso, leading to stretching in one plane at the time. Using GI, the attempt was to improve the thoracic range of motion by using breathing exercises with stretching in a more functional way, expanding the thorax in multiple planes at the same time. There are some studies in AS presenting different breathing exercises and their effects on lung function parameters [25–28], however, none resembling the GI technique.

Some of the participants reported temporary symptoms such as tension in the chest and fatigue. Such symptoms have been reported in earlier studies [11–13]. However, one patient had extreme dizziness and local paresthesia during the postintervention assessment. Dizziness might depend on a reduction in preload as a result of the increased intrathoracic pressure when the participants perform GI [8]. Earlier, cases with neurological symptoms during GI probably due to arterial gas embolism (AGE) have been reported [29]. The patient in the present study showed neurological symptoms and was assessed by a physician, explaining the symptoms to be caused by hyperventilation, but the distribution of symptoms correlates better with a transient ischemic attack or the above suggested AGE. Neither of these three diagnoses could be validated at the time; however, this must be more carefully studied in patients especially in assessment situations, when some patients might be prone to maximal exertion during GI.

## 5. Conclusion

No definite conclusions can be drawn from this pilot investigation, but GI might have the potential to increase thoracic range of motion in patients with AS. If learned properly, GI exercise can increase VC in patients with AS. As diffuse neurological symptoms appeared in one patient, it is important to investigate the effects further.

## Figures and Tables

**Table 1:** Baseline characteristics of the 6 subjects fulfilling the study protocol.

	Median (range)
Gender, male/female (*n*)	6/0
Age, years	42 (26–55)
Disease duration, years	14 (0.5–28)
Smoking, yes/no (*n*)	0/6
Weight, kg	92.5 (81–106)
Height, cm	180.5 (177–189)
BMI^1^, kg/m^2^	28.1 (24.9–30.0)
VC^2^ % of predicted	96 (67–102)
PEF^3^ % of predicted	102.5 (90–113)

^1^Body mass index (BMI), ^2^vital capacity (VC), and ^3^peak expiratory flow (PEF).

**Table 2:** Individual results from baseline (pre) and follow-up after 12 weeks (post) of Glossopharyngeal Insufflation for the six patients fulfilling the study protocol.

	Case 1		Case 2		Case 3		Case 4		Case 5		Case 6	
	Pre	Post	Pre	Post	Pre	Post	Pre	Post	Pre	Post	Pre	Post
VC^1^, L	4.84	5.18	5.63	4.94	3.68	3.95	3.11	3.14	5.79	5.69	5.56	5.89
PEF^2^, L/min	557	605	545	539	561	528	589	559	586	491	625	656
C7-30, cm	5.0	5.5	6.0	5.5	3.5	3.5	0.5	1.0	3.0	—	2.0	4.0
Tragus-wall, cm	10	9.5	11.5	11.5	11.5	11.0	18	17.0	10.5	—	16	13..5
BX^3^ diff, cm	7.0	8.5	6.0	7.5	2.0	4.0	4.0	5.0	6.0	—	7.0	9.5
BC^4^ diff, cm	9.5	11.5	4.5	6.5	3.0	3.5	2.0	2.0	4.0	—	6.0	8.0
BAS DAI^5^, 0–10	3.4	3.4	0.4	1.3	1.8	2.0	1.5	2.3	2.3	—	0.7	0.8
BASFI^6^, 0–10	0.6	0.25	0.2	0.7	1.5	2.0	1.0	1.0	1.1	—	2.1	1.9
BASG1^7^, 0–10	1.6	2.4	0.4	2.6	2.5	2.3	0.2	0.3	1.9	—	0.6	1.2
VCGI^8^ (L)		6.75		5.40		4.05		3.57		5.76		7.78
VC increase with GI (%)		30.3		1.4		2.5		13.7		1.2		32.1

^1^Vital capacity (VC), ^2^peak expiratory flow (PEF), ^3^level of xiphoid process on the chest wall (BX) difference of inhalation and exhalation, ^4^level of the fourth costae on the chest wall (BC) difference of inhalation and exhalation, ^5^the Bath Ankylosing Spondylitis Disease Activity Index (BASDAI), ^6^the Bath Ankylosing Spondylitis Functional Index (BASFI), ^7^the Bath Ankylosing Spondylitis Global Index (BASG1), and ^8^vital Capacity using Glossopharyngeal Insufflation (VCGI).
